# Supplementary material for: Influence of Vineyard Location, Cluster Thinning and Spontaneous Alcoholic Fermentation on Wine Composition
Source: Foods. 2025 Mar 22;14(7):1101. doi: 10.3390/foods14071101 (PMC11989029; doi:10.3390/foods14071101)
Supplement: Supplementary file 1 [file foods-14-01101-s001.zip › foods-3517928-supplementary.pdf]

Table S1. Date, day of the year and growing degree days (GDD) accumulation for phenological stages of Merlot variety at the location M for 2020 and 2021 vintages.

	**BBCH keys**		**Date**		**Day of the year**		**GDD** [1]	
Phenological stages and BBCH identification keys [38]	2020	2021	2020	2021	2020	2021	2020	2021
Fruit set (BBCH 71-79)	71-73	/ [2]	26.6.	/	178	/	479	/
	79	75	21.7.	26.7.	203	207	737	814
Ripening of the berries (BBCH 81-88)	83	81	24.8.	24.8.	237	236	1154	1160
	/	88	/	21.9.	/	264	/	1369
Harvest (BBCH 89)	89	89	6.10.	6.10.	280	279	1488	1456

[1] GDD calculated from 1st April (base T>10°C) until harvest. [2] Phenological stages was not recorded, therefore day of the year and GDD not calculated.

Table S2. Date, day of the year and growing degree days (GDD) accumulation for phenological stages of Merlot variety at location B for 2020 and 2021 vintages.

	**BBCH keys**		**Date**		**Day of the year**		**GDD** [1]	
Phenological stages and BBCH identification keys [38]	2020	2021	2020	2021	2020	2021	2020	2021
Leaf development and inflorescence emerge (BBCH 1./5.)	18/57	15/53	26.5.	26.5.	147	146	264	165
Fruit set (BBCH 71-79)	79	78-79	20.7.	14.7.	202	195	769	740
Ripening of the berries (BBCH 81-88)	83	81	21.8.	24.8.	234	236	1192	1278
	88	88	10.9.	3.9.	254	246	1406	1351
Harvest (BBCH 89)	89	89	30.9.	5.10.	274	278	1574	1628

[1] GDD calculated from 1st April (base T>10°C) until harvest.

Table S3. Viticultural parameters at both locations (M, B) and crop loads (C, CT) in both vintages (2020, 2021).

	Vintage/Crop loads (C, CT)[1]				*p* values[2]		
**LOCATION M**	2020		2021				
	M-C	M-CT	M-C	M-CT	T	V	T×V
Shoots/m	8.7±1.2	8.3±1.4	8.6±1.8	7.4±1.7	ns	ns	ns
Clusters/vine before thinning	13.5±2.5	13.1±2.0	12.8±2.1a	9.7±2.4b	**	***	*
Clusters/vine at harvest	13.2±2.5a	9.1±1.1b	13.7±2.4a	5.6±1.6b	***	ns	*
Bunch weight (g)	203.1±37.6	192.0±32.3	182.8±18.3	204.5±35.6	ns	ns	ns
Yield/vine (kg)	2.66±0.66a	1.72±0.23b	2.50±0.45a	1.15±0.41b	***	ns	ns
**LOCATION B**	2020		2021				
	B-C	B-CT	B-C	B-CT	T	V	T×V
Shoots/m	8.2±1.9	8.2±1.7	8.3±0.9	8.0±1.6	ns	ns	ns
Clusters/vine before thinning	14.6±2.6a	14.4±2.2ab	12.6±2.9bc	11.1±3.5c	ns	***	ns
Clusters/vine at harvest	14.6±2.7a	10.7±0.7b	11.6±2.9ab	7.3±1.3c	***	***	ns
Bunch weight (g)	222.8±37.9	219.8±37.0	143.9±32.4	134.9±40.5	ns	***	ns
Yield/vine (kg)	3.18±0.43a	2.31±0.28b	1.65±0.50c	0.98±0.37d	***	***	ns

[1] ANOVA was used to compare data. Means followed by a different letter in a row are significant at $p \leq 0.05$ (Fisher's LSD). All reported uncertainties are standard deviations of n=18 or n=20 (location M) and n=17 or n=19 (location B) replicates per treatment (see Materials and methods for further details). [2] The asterisks indicate the level of significance of two-way ANOVA for T treatment, V, vintage and T×V, interaction treatment*vintage: * $p \leq 0.05$, ** $p \leq 0.01$ and *** $p \leq 0.001$, whereas ns indicates no significant differences.

Table S4. Berries fresh mass and must parameters at both locations (M, B) and crop loads (C, CT) in both vintages (2020,2021).

	Vintage/Crop load (C, CT)[1]						
**LOCATION M**	2020[3]		2021		*p* values[2]		
	M-C	M-CT	M-C	M-CT	T	V	T×V
100 berries fresh mass (g)	203±11	186±8	175±7	185±9	ns	*	*
TSS (°Bx)	20.17±0.78	21.07±0.25	21.47±0.06	21.70±0.00	ns	**	ns
TAc (g/L)	5.5±0.4	4.9±0.3	6.4±0.1	6.2±0.0	ns	***	ns
pH	3.46±0.08	3.50±0.07	3.20±0.01	3.22±0.01	ns	***	ns
**LOCATION B**	2020		2021				
	B-C	B-CT	B-C	B-CT	T	V	T×V
100 berries fresh mass (g)	193±15	195±7	186±8	188±8	ns	ns	ns
TSS (°Bx)	22.97±0.47	23.88±0.47	24.77±0.06	25.17±0.06	ns	ns	ns
TAc (g/L)	6.5±0.2	6.5±0.4	4.6±0.0	4.5±0.2	ns	ns	ns
pH	3.40±0.00	3.47±0.04	3.40±0.02	3.44±0.02	ns	ns	ns

[1] Two-way ANOVA was used to compare data. All stated uncertainty is a standard deviation of three replicates per treatment. [2] The asterisks indicate the level of significance of two-way ANOVA for T treatment, V, vintage and T×V, interaction treatment*vintage: * $p \le 0.05$, ** $p \le 0.01$ and *** $p \le 0.001$, whereas ns indicates no significant differences.

Table S5. Reducing sugar (RS), malic acid and lactic acid concentrations in experimental Merlot wines from two locations (M, B), two crop loads (C, CT) and two vintages (2020, 2021) after finished inoculated (iAF) and spontaneous (sAF) alcoholic fermentation.

**LOCATION M**	RS (g/L)	Malic acid (g/L)	Lactic acid (g/L)
2020			
M-C	1.1±0.1[1]	<0.10	1.93±0.03
M-CT	1.1±0.1	<0.10	2.01±0.14
M-C_sAF	1.4±0.4	<0.10	2.12±0.23
2021			
M-C	1.4±0.3	<0.10	1.98±0.05
M-CT	1.2±0.2	<0.10	1.90±0.04
M-C_sAF	1.1±0.1	<0.10	2.20±0.11
**LOCATION B**			
2020			
B-C	1.2±0.1	<0.10	2.23±0.05
B-CT	1.4±0.1	<0.10	2.18±0.07
2021			
B-C	1.2±0.1	<0.10	1.19±0.03
B-CT	1.8±0.2	<0.10	1.31±0.04
B-C_sAF	1.1±0.1	<0.10	1.08±0.04

[1] All stated uncertainty is a standard deviation of three replicates per treatment.

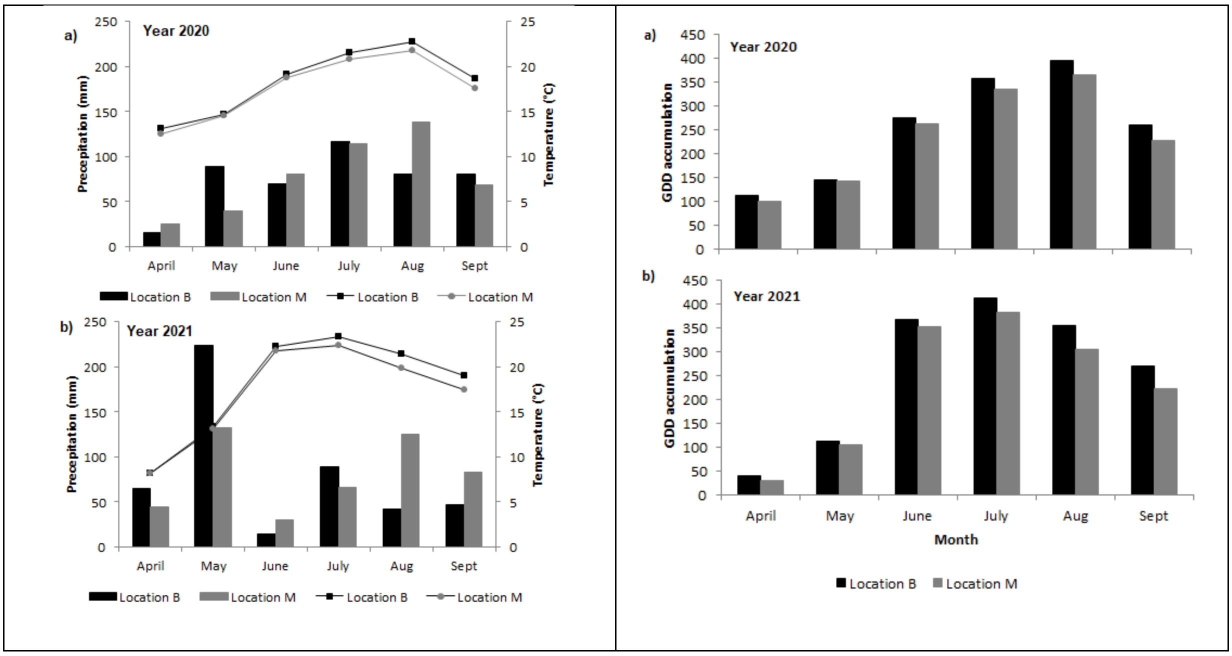

Figure S1. Monthly average temperature (lines) and precipitation (histograms) (left graph) and monthly growing degree days (GDD) (right graph) for vintages 2020 (a) and 2021 (b), at the locations B and M.

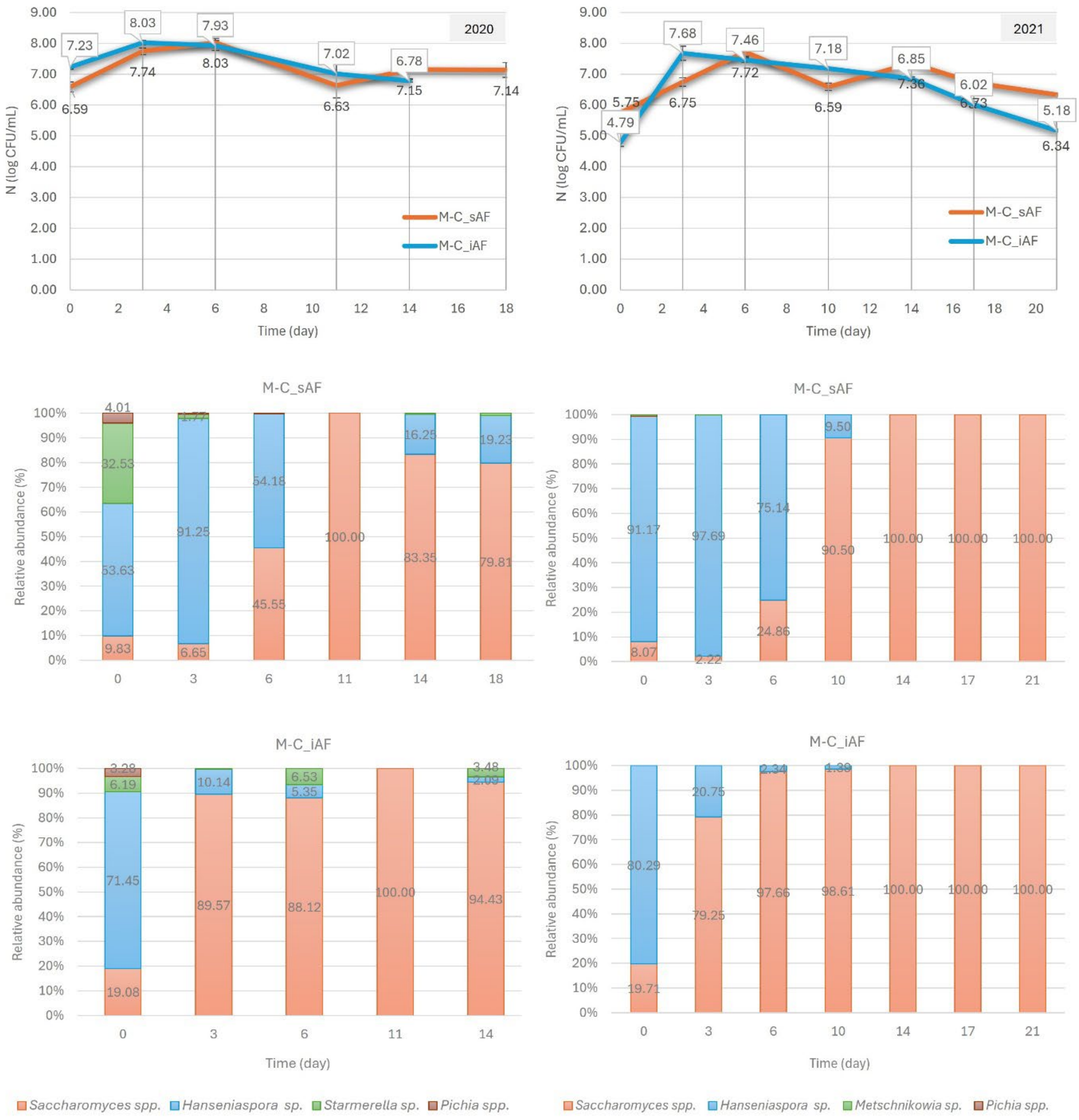

Figure S2. Yeast cell concentration (log CFU/mL) (upper plots; the mean values and standard deviations of three repetitions per treatment are displayed) and yeast population dynamics during spontaneous (M-C_sAF) and inoculated (M-C_iAF) alcoholic fermentations of Merlot mash from location M (middle and lower plots; the mean values of three repetitions and the values for the relative abundance above 1% are shown; vintage 2020-left, vintage 2021-right.

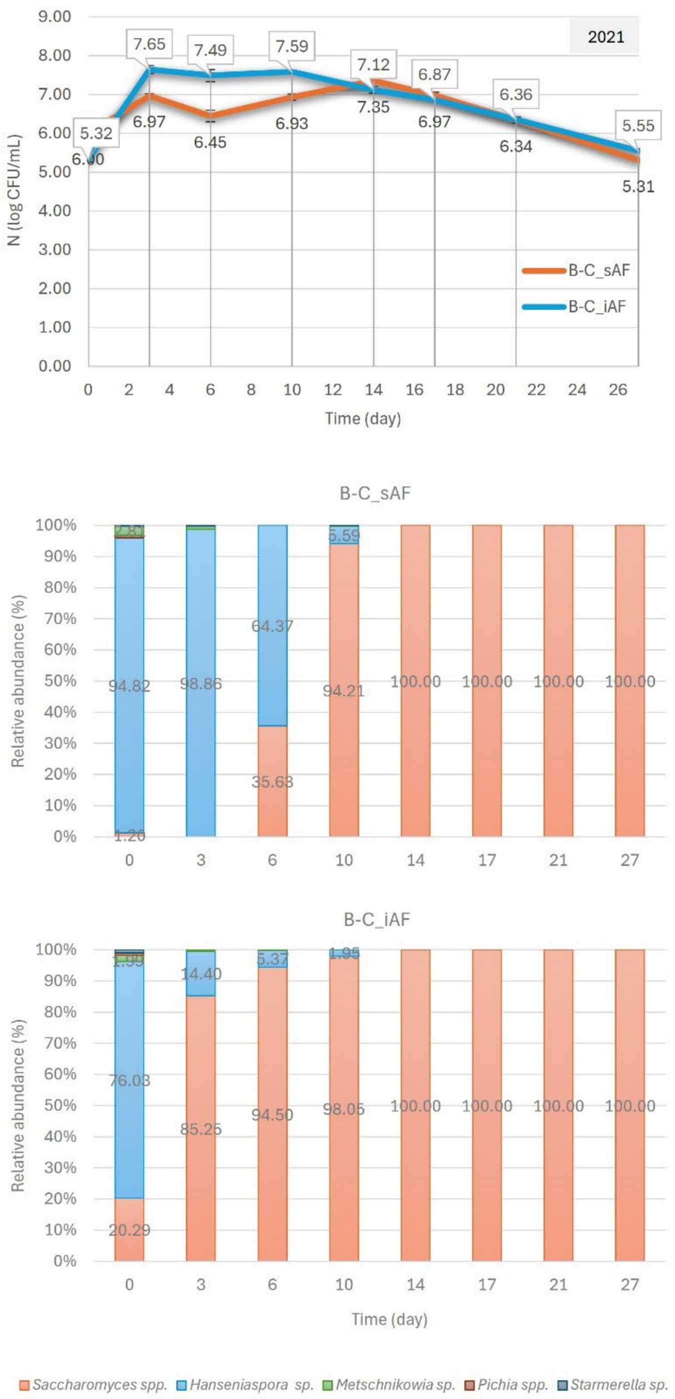

Figure S3. Yeast cell concentration (log CFU/mL) (upper plot; the mean values and standard deviations of three repetitions per treatment are displayed) and yeast population dynamics during spontaneous (B-C_sAF) and inoculated (B-C_iAF) alcoholic fermentations of Merlot mash from location B in 2021 (middle and lower plots; the mean values of three repetitions and the values for the relative abundance above 1% are shown).
